# On Some of the Uses of Carbolic Acid

**Published:** 1873-03

**Authors:** J. F. M'Donald

**Affiliations:** Hopewell, Nova Scotia.


### ARTICLE VII.

### *On some of the Uses of Carbolic Acid.*

BY J. F. M'DONALD, M. D., HOPEWELL, NOVA SCOTIA.

In the *Medical News* for December, 1872, I noticed an article on the Local Treatment, by Carbolic Acid, of Diphtheria, by Dr. H. Reynolds, of Maine. I wish to add my tesimony to the use of carbolic acid in the treatment of that disease.

About four years ago I began to use carbolic acid in the treatment of inflammatory sore throat and tonsillitis, in which I found it very useful. I use a gargle of a weak solution of carbolic acid, with chlorate of potassa. In severe cases I apply, by means of a camel hair brush or wisp of cotton, a solution of the acid and water, equal parts.

In October, 1870, I first used carbolic acid in the treatment of diphtheria, and have, thus far, found it eminently successful. I apply, by means of a camel-hair pencil or cotton wisp, to the part affected, carbolic acid 15 parts, water 5 parts, or equal parts of each. I also use a gargle of a solution of the acid and chlorate of potassa.

In toothache it acts like a charm; in most cases relieving pain almost instantaneously. I apply the pure acid on lint to the carious tooth, repeating, if necessary, till pain is relieved. The acid kept in solution by adding one-twentieth of its bulk of water is preferable. It will not injure the sound teeth.

I have treated some skin diseases successfully by means of carbolic acid. In scabies I never knew it fail. I have found it safe, and not unpleasant. A professional friend told me a year ago that "Carbolic acid was his specific in treatment of itch."

In herpes, eczema, tinea, psoriasis, and acne, I have found it very useful. In eczema infantilis it is especially useful. I have never seen it fail in producing a speedy cure.

The solutions I generally use are—acid carbol. ℨ j, or ℨ ij, aq. ad. Oj, or, what is better, the acid dissolved in glycerine. In tinea tonsurans I apply, by means of a camel hair pencil, acid carbol. 15 parts, water 5 parts; it will rarely need a second application.

Internally I have used carbolic acid, but cannot say that I have seen any benefit from its use. In nausea and vomiting of pregnancy, in my hands, it has been a failure.—*Medical News.*

